# Factors Influencing Contrast Sensitivity Function in Myopic Eyes

**DOI:** 10.1371/journal.pone.0113562

**Published:** 2014-11-17

**Authors:** Kazutaka Kamiya, Kimiya Shimizu, Ayaka Iijima, Hidenaga Kobashi

**Affiliations:** Department of Ophthalmology, University of Kitasato School of Medicine, Sagamihara, Kanagawa, Japan; Medical College of Soochow University, China

## Abstract

**Purpose:**

To evaluate the factors affecting the area under the log contrast sensitivity function (AULCSF) in healthy myopic eyes.

**Methods:**

We retrospectively examined 201 eyes of 201 consecutive subjects (age, 31.8±7.4 years (mean ± standard deviation)) with myopic refractive errors of −1.25 to −8.25 diopters (D). From the contrast sensitivity data, the area under the log contrast sensitivity function (AULCSF) was calculated. Stepwise multiple regression analysis was used to assess the factors affecting the AULCSF.

**Results:**

The mean AULSCF was 1.09±0.09 (0.89 to 1.55). Explanatory variables relevant to the AULCSF were, in order of influence, the objective scattering index (OSI) (p = 0.018, partial regression coefficient B = –0.032) and logMAR CDVA (p = 0.022, B = –0.209) (adjusted R^2^ = 0.231). No significant correlation was seen with other clinical factors such as gender, manifest refraction, pupil size, lens density, corneal HOAs, or ocular HOAs.

**Conclusions:**

Although the great majority of the variance remains unexplained, eyes with lower OSI and better CDVA are more predisposed to show higher contrast sensitivity function. These results indicate that not only CDVA but also intraocular forward scattering may play some role in predicting the contrast sensitivity function in myopic subjects.

## Introduction

Optical aberrations and light scatter can lead to the degradation of image quality on the retina, resulting in the deterioration of subjective visual performance. Contrast sensitivity testing has been shown to be clinically useful for examining the subtle changes in subjective visual performance. [1], [2] However, this test cannot determine whether the changes originated from optical aberrations or light scatter. Accordingly, it is of importance to assess individually the roles of optical aberrations and light scatter in the contrast sensitivity function in a clinical setting. However, as far as we can ascertain, no detailed report on the clinical factors behind the contrast sensitivity function in an ophthalmologically normal population has as yet appeared. Moreover, no relationship between the contrast sensitivity function and intraocular scattering in such subjects has so far been quantitatively elucidated. The purpose of this study is to retrospectively investigate the clinical factors that influence the contrast sensitivity function using multivariate regression analysis in a large cohort of healthy but myopic subjects.

## Materials and Methods

### Study Population

This retrospective review of data was approved by the Institutional Review Board at Kitasato University and followed the tenets of the Declaration of Helsinki. Our Institutional Review Board waived the requirement for informed consent for this retrospective study. Patient data was anonymized before access and/or analysis. Two hundred one eyes of the 201 consecutive subjects (73 men and 128 women), who were examined at Kitasato University Hospital for refractive consultation, and who had no ocular diseases (corneal disease, clinically apparent cataract, or retinal or optic nerve pathology), except for myopic refractive errors (myopia and myopic astigmatism), were included in this retrospective observational study. Only one eye per subject was selected randomly for statistical analysis. All eyes had corrected distance visual acuity (CDVA) of 20/20 or better. Eyes with keratoconus were excluded from the study by using the keratoconus screening test of Placido disk videokeratography (TMS-2, Tomey, Nagoya, Japan).

### Assessment of Visual Acuity, Refraction, and Contrast Sensitivity Function

Visual acuity measurement was performed by experienced optometrists using a Snellen chart with Japanese letters at a distance of 5 m with best correction (but not with habitual correction). Refraction was measured by an optometrist using an automated refractometer (ARK-700A, Nidek, Gamagori, Japan), and the results were used as a starting point for a full subjective and manifest refraction. Contrast sensitivity function was measured by a contrast sensitivity unit (VCTS-6500, Vistech) under photopic conditions (500 lux). The test was performed with best spectacle correction at 2.5 m. From the contrast sensitivity, the area under the log contrast sensitivity function (AULCSF) was determined as described previously. [3] In brief, the log of contrast sensitivity was plotted as a function of log spatial frequency, and third-order polynomials were fitted to the data. The fitted function was integrated between the fixed limits of log spatial frequencies of 0.18 (corresponding to 1.5 cycles/degree) and 1.26 (corresponding to 18 cycles/degree), and the resultant value was defined as the AULCSF.

### Assessment of Pupil Size, Backward and Forward Scatterings, and Higher-Order Aberrations

The density of the crystalline lens, as a measure of backward scattering of the crystalline lens, was determined by the rotating Scheimpflug imaging system (Pentacam HR, Oculus, Wetzlar, Germany). The Scheimpflug imaging device provides an image of the whole lens and an objective measurement of the lens density at the chosen point on a scale of 0 to 100 (0 = no cloudiness; 100 = completely opaque lens). [4], [5] On the 3-dimensional plot of the anterior segment, each section of which runs through the corneal vertex, the required lens density was taken as the peak value of the area of the nucleus on the image in the horizontal plane (0 to 180 degrees). Pupil size was also measured by the rotating Scheimpflug imaging system under the same light conditions as those in which the patients were resting in order to reduce the individual changes in pupil size. Because of the brightness of the light source, measurements under dark conditions were not possible. Video images were captured, and the entrance pupil diameter was measured automatically with digital infrared pupillometry.

The objective scattering index (OSI), as a measure of intraocular forward scattered light, was determined by an Optical Quality Analysis System™ (OQAS; Visiometrics, Terrassa, Spain), which is designed on the basis of the asymmetric pattern of the double-pass technique, for a 4-mm pupil. The index is calculated by evaluating the amount of light outside the double-pass retinal intensity PSF image in relation to the amount of light on the center. In the particular case of the instrument OQAS, the central area selected was a circle of a radius of 1 minute of arc, while the peripheral zone was a ring set between 12 and 20 minutes of arc. [6] The OSI for normal eyes would range around 1, while values over 5 would represent highly scattered systems.

Corneal and ocular higher-order aberrations (HOAs) for a 4-mm pupil were measured by Hartmann-Shack aberrometry (KR-9000, Topcon, Tokyo, Japan). The root-mean-square of the third-order Zernike coefficients was utilized to represent third-order aberrations, the root-mean-square of the fourth-order coefficient to represent fourth-order aberrations. Total HOAs were calculated as the root-mean-square of the third- and fourth-order coefficients. All examinations were performed by experienced ophthalmic technicians.

### Statistical Analyses

Stepwise multiple regression analysis was performed to investigate the relation between several variables and the contrast sensitivity function. The dependent variable was the AULCSF. The explanatory variables included patient age, gender, logarithm of the minimal angle of resolution (logMAR) of CDVA, manifest refraction, pupil size, lens density, OSI, and corneal and ocular HOAs. Spearman’s rank correlation test was also performed to assess the relationships of the AULCSF with other variables. All statistical analyses were performed using a commercially available statistical software (Ekuseru-Toukei 2010, Social Survey Research Information Co, Ltd., Tokyo, Japan). The results are expressed as mean ± standard deviation, and a P-value less than 0.05 was considered statistically significant.

## Results

**Figure 1:**
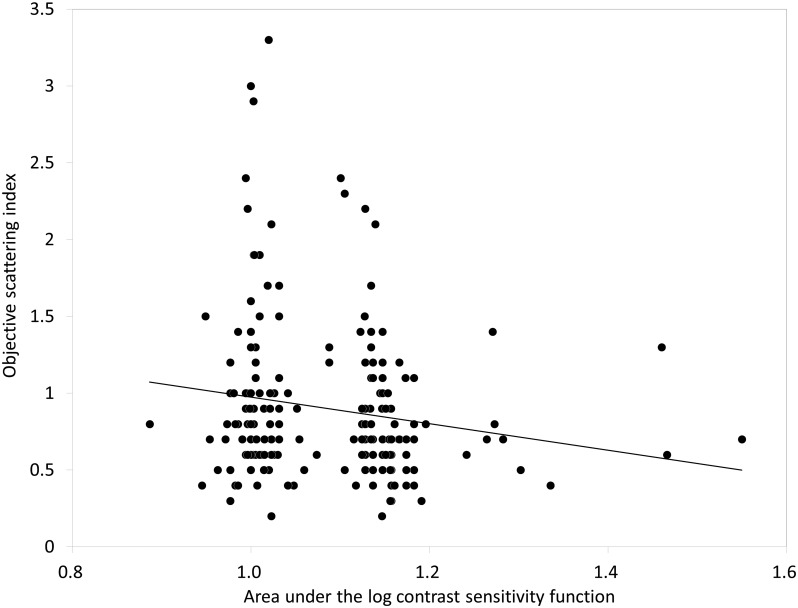
Correlation between the area under log contrast sensitivity function and the objective scattering index.

**Figure 2:**
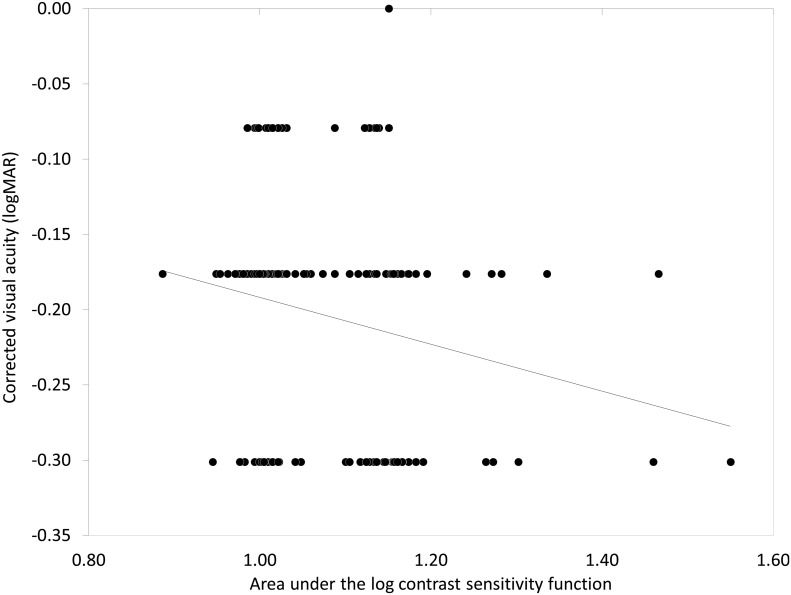
Correlation between the area under log contrast sensitivity function and corrected distance visual acuity.

The demographics of the study population are shown in Table 1. The mean AULSCF was 1.09±0.09 (0.89 to 1.55). The results of multiple regression analysis are shown in Table 2. The explanatory variables relevant to the AULSCF were the OSI (p = 0.018, partial regression coefficient B = –0.032) and logMAR CDVA (p = 0.022, B = –0.209) (adjusted R^2^ = 0.231). The multiple regression equation was expressed as follows: AULCSF = (–0.032×OSI)+(–0.209×logMAR CDVA)+1.034. There was no significant correlation shown with other clinical factors such as age, gender, manifest refraction, pupil size, lens density, corneal HOAs, or ocular HOAs. The standardized partial regression coefficient was calculated in order to determine the magnitude of each variable’s influence. The OSI was the most relevant variable, and logMAR CDVA was the second. Similar results were obtained by Spearman’s rank correlation test as shown in Table 2. The relationships of the AULCSF with the OSI and with logMAR CDVA are shown in Figures 1 and 2, respectively. With lower OSI, better CDVA, or both, the AULSCF was significantly increased in myopic subjects.

**Table 1:** Demographics of the study population.

Patient Demographics	
Age (years)	31.8±7.4 years (range, 18 to 53 years)
Gender (Male:Female)	M:F = 73:128
LogMAR CDVA	−0.21±0.07 (range, −0.30 to 0.00)
Manifest spherical equivalent (D)	−4.50±1.73 D (range, −1.25 to −8.25 D)
Pupil size (mm)	2.90±0.45 mm (range, 1.87 to 4.76 mm)
Lens density	7.62±2.30 (range, 5.1 to 17.5)
Corneal HOAs (µm)	0.09±0.04 µm (range, 0.03 to 0.51 µm)
Ocular HOAs (µm)	0.05±0.02 µm (range, 0.02 to 0.11 µm)
OSI	0.90±0.50 (range, 0.20 to 3.30)

D = diopter, logMAR = logMAR = logarithm of the minimal angle of resolution, CDVA = corrected distance visual acuity, HOAs = higher-order aberrations, OSI = objective scattering index.

**Table 2:** Results of correlation analysis and stepwise multiple regression analysis to select variables relevant to the area under the log contrast sensitivity function in myopic subjects.

Variables	Spearmancorrelationcoefficient	P value	Partialregressioncoefficient		Standardized partialregressioncoefficient	P value
Objective scattering index	−0.193	0.006	−0.032		−0.172	0.018
LogMAR CDVA	−0.206	0.003	−0.209		−0.163	0.022
Age (years)	−0.030	0.677	not included			-
Gender (male = 0, female = 1)	0.080	0.440	not included			-
Manifest refraction (D)	−0.055	0.257	not included			-
Pupil size (mm)	0.051	0.472	not included			
Lens density	0.020	0.777	not included			
Corneal HOAs (µm, 4 mm)	0.078	0.268	not included			-
Ocular HOAs (µm, 4 mm)	−0.049	0.486	not included			-
			1.034	Constant		Adjusted R^2^ = 0.231

logMAR = logarithm of the minimal angle of resolution, CDVA = corrected distance visual acuity, D = diopter, HOAs = higher-order aberrations.

## Discussion

Although the OSI and CDVA alone cannot provide sufficient explanation, as evidenced by the small R^2^ value (R^2^ = 0.231), both the OSI and CDVA can affect the contrast sensitivity function in an ophthalmologically normal population. As far as we can ascertain, this is the first published study to assess the detailed background factors influencing the contrast sensitivity function using multiple regression analysis in a large cohort of healthy subjects. Since contrast sensitivity is known to be influenced by multiple factors, including retina and brain processing, [7], [8] it is understandable that the optics alone cannot fully account for the contrast sensitivity. The increase in ocular forward scatter as a result of the changes in the transparency of the ocular tissues, especially those of the crystalline lens, may play some role in the decrease in contrast sensitivity. We should be aware that eyes with lower intraocular forward scattering and eyes with better visual acuity are more predisposed to show higher contrast sensitivity in myopic healthy subjects.

Visual acuity encompasses a very small central visual angle (0.02 degrees), whereas contrast sensitivity encompasses an angle of approximately 0.30 degrees. It is reasonable that CDVA was significantly correlated with the AULSCF in the current study, a fact suggesting that CDVA is one of the most important metrics to assess overall visual performance for clinical use. It has been demonstrated that contrast sensitivity was decreased with aging. [9] Contrary to our expectations, we found no significant association between the AULCSF and subject age in the current study. It may be that the range of the subjective age was relatively narrow, and that older patients (age>53 years), often affected with age-related cataracts, were not included in the current study, as evidenced by the relatively low lens density values (7.62±2.30). It has also been demonstrated that the contrast sensitivity function is affected by pupil size. [10]–[12] However, we also found no significant correlation between AULSCF and pupil size. It may be attributed to a slight difference of luminance conditions between the AULCSF and pupil size measurements. Further studies with a wide range of subject ages under different light conditions are still necessary in order to clarify these points.

With regard to intraocular scattering and HOAs, we found a significant correlation of the AULSCF with the OSI, but not with the lens density or HOAs in the present study, suggesting that the forward light scattering plays a more essential role in the contrast sensitivity function than the backward scattering or HOAs in myopic subjects. It has been reported that contrast sensitivity could be clearly compromised due to intraocular scattering, even in photopic conditions. [13], [14] Lee et al showed that the OSI was positively correlated with visual acuity, but not with contrast sensitivity at any spatial frequencies. [15] Hennelly et al reported that the aging eye after 45 years of age showed a more rapid increase in forward scatter, accompanied by a reduction in contrast sensitivity, despite apparently good visual acuity. [16] Fujikado et al demonstrated that loss of contrast sensitivity was predominantly due to backward light scattering and to HOA in eyes with nuclear cataract, but also resulted from forward light scattering and HOA in eyes with cortical cataract. [17] Pérez et al explored the combined effect of light scattering and HOA on visual performance, and showed that contrast sensitivity was reduced less by scattering when spherical aberration was present as compared with the cases without spherical aberration. [18] To date, there have been several studies assessing the effect of HOAs on the contrast sensitivity function in normal and diseased eyes. [17]–[21] Liang et al showed that human eye aberrations play a crucial role in degrading retinal image quality. [19] Oshika et al reported that the AULCSF was significantly correlated with coma-like aberrations, but not spherical-like aberrations. [20] Feizi et al demonstrated that the AULSCF was significantly associated with total HOAs and fourth-order aberrations, but not third-order aberrations, in myopic eyes. [21] Fujikado et al found a significant association between the AULCSF and HOAs in eyes with cataract. [17] Although we cannot fully explain the discrepancy between the current and previous findings, the sample size, the methodology of the measurements, the distribution of subject age, the presence of age-related cataract, and other subject backgrounds of the study population, may contribute to this discrepancy.

There several limitations to this study. Firstly, it was conducted in a retrospective fashion. A randomized, controlled study may provide further information for confirming the authenticity of these results. Secondly, we included only myopic subjects who were examined for refractive consultation. Thirdly, we determined the contrast sensitivity function using the VCTS-6500 only under photopic conditions. The Vistech contrast sensitivity charts have been reported to have poor test-retest repeatability in patients with normal vision and early or subtle eye disease [22] and in cataractous and post-LASIK patients, [23] and there is the possible risk of a “ceiling effect” in that the dynamic range of the test is insufficient for people with good vision in this study, [23] although it is widely used in a clinical setting. Further studies on the contrast sensitivity function under photopic and mesopic conditions in eyes with a wide range of refraction are necessary in order to accurately evaluate visual function in depth.

In conclusion, our study on the factors influencing contrast sensitivity may support the view that eyes with lower OSI and eyes with better CDVA showed higher contrast sensitivity in an ophthalmologically normal population, although the great majority of the variance remained unexplained. According to our experience, intraocular forward scattering may play a more vital role in subjective visual performance in myopic healthy subjects than backward scattering or HOAs. Not only visual acuity but also intraocular forward scattering should be taken into consideration for predicting the contrast sensitivity function in such subjects.
